# Caregiver-reported oral health-related quality of life in children with Mowat–Wilson syndrome: a comparative study

**DOI:** 10.3389/fdmed.2026.1953280

**Published:** 2026-10-09

**Authors:** Silvia Cirio, Claudia Salerno, Aesha Allam, Giulia Zambon, Araxi Balian, Maria Grazia Cagetti

**Affiliations:** 1 Department of Biomedical, Surgical and Dental Sciences, University of Milan, Milan, Italy; 2 Dental and Stomatology Unit, Surgical Area Department, Cittadella Hospital, Cittadella, Italy

**Keywords:** autism, Mowat–Wilson syndrome, oral health-related quality of life, special health care needs, unmet dental needs

## Abstract

**Background:**

Mowat–Wilson syndrome (MWS) is a rare genetic disorder characterized by intellectual disability, distinctive craniofacial features, behavioral impairments, and congenital anomalies. Although oral and craniofacial abnormalities are frequently reported, their impact on oral health-related quality of life (OHRQoL) has not been investigated. This study aimed to evaluate OHRQoL in children with MWS and compare it with peers with autism spectrum disorder (ASD) and neurotypical controls. Secondary analyses explored caregiver functional limitations and dental care characteristics.

**Methods:**

A cross-sectional questionnaire-based study was conducted involving caregivers of children aged 6–18 years with MWS (*n* = 19), ASD (*n* = 19), and neurotypical controls (*n* = 38). OHRQoL was assessed using the short-form Parental-Caregiver Perception Questionnaire (*P*-CPQ) and Family Impact Scale (FIS). Additional questions explored socioeconomic characteristics, dental care setting and attendance, and parental perceptions of oral health. Group comparisons were performed using non-parametric and parametric tests, with significance set at *p* < 0.05.

**Results:**

Children with MWS showed significantly higher total P-CPQ scores than neurotypical controls (37.11 ± 8.65 vs. 26.23 ± 5.05; *p* < 0.001), indicating poorer OHRQoL. The greatest impairments were observed in the functional limitation domain, particularly regarding chewing difficulties, mouth breathing, slower eating, and sleep disturbances (all *p* < 0.001). Halitosis and food impaction on the palate were also significantly more frequent in the MWS group. Parents of children with MWS reported a greater impact of oral conditions on wellbeing than both ASD and neurotypical groups (*p* < 0.05). Although OHRQoL impairment was comparable between MWS and ASD participants, ASD was associated with greater family burden, reflected by higher FIS scores (16.42 ± 7.09 vs. 21.58 ± 4.85; *p* = 0.016). Both MWS and ASD groups exhibited higher combined P-CPQ + FIS scores than neurotypical controls (*p* < 0.01).

**Conclusions:**

Both MWS and ASD were associated with poorer OHRQoL than neurotypical status, although distinct patterns of impairment emerged. MWS was primarily characterized by functional oral limitations and a greater impact of oral conditions on wellbeing, whereas ASD was associated with greater psychosocial and family burden. These findings support the existence of a syndrome-specific profile of caregiver-perceived unmet oral health needs and functional limitations in children with complex healthcare conditions and emphasize the importance of individualized, multidisciplinary dental care strategies.

## 1 Introduction

Oral health is a primary component of general health and well-being (1). Its impairment may result in pain, discomfort, acute and chronic infections, impaired nutrition and sleep, and reduced social functioning (2). In children, poor oral health may additionally contribute to school absenteeism and negatively affect educational attainment and academic performance. Although oral diseases are highly prevalent in the general population, they occur more frequently among individuals with special healthcare needs, particularly those with intellectual and developmental disabilities (3). In rare neurodevelopmental syndromes, unmet dental needs may not be limited to untreated disease, but may also include undetected oral functional impairment, feeding-related problems, delayed referral to specialized dental services, and lack of syndrome-specific preventive pathways. In addition, difficulties in maintaining oral hygiene, behavioral and communication impairments, limited cooperation during dental procedure may significantly worsen oral health status and its impact on daily life. Within this framework, oral health–related quality of life (OHRQoL) represents a multidimensional construct encompassing the impact of oral health on functional, psychological, emotional, and social well-being. OHRQoL includes aspects related to oral symptoms, chewing ability, sleeping, self-esteem, interpersonal relationships, school performance, and family functioning (4). OHRQoL has been recognized by the World Health Organization as a key component of global oral health strategies (5).

Several validated instruments have been developed to assess OHRQoL across different age groups and populations (6). Among younger children, the Early Childhood Oral Health Impact Scale (ECOHIS) is one of the most widely used questionnaires (7), whereas in older children and adolescents OHRQoL is commonly evaluated through instruments such as the Parental-Caregivers Perception Questionnaire (P-CPQ) and the Family Impact Scale (FIS), which investigate the impact of oral conditions on both affected individuals and their families. OHRQoL has become an increasingly relevant patient-centered outcome measure, as it evaluates not only oral symptoms and functional limitations, but also the psychological, social, and familial consequences associated with oral conditions (8, 9).

Mowat–Wilson syndrome (MWS) is a rare genetic disorder first described by D.R. Mowat and M.J. Wilson in 1998 (10). It is characterized by distinctive facial dysmorphism, moderate to severe intellectual disability, microcephaly, epilepsy, and a broad spectrum of congenital anomalies, including Hirschsprung disease (HSCR), urogenital and renal abnormalities, congenital heart defects (CHD), agenesis of the corpus callosum (ACC), and ocular defects (11). The prevalence of MWS is currently unknown, although it has been estimated to range between 1 in 50,000 and 1 in 70,000 live births (12). More than 300 patients have been reported worldwide; however, the syndrome is likely underdiagnosed (13). The male-to-female ratio is approximately 1.42:1 (14, 15). MWS is caused by heterozygous loss-of-function mutations in the ZEB2 (Zinc Finger E-box-Binding Homeobox 2) gene located on chromosome 2q22.3 (16, 17). Facial dysmorphism, observed in more than 90% of affected individuals, is characterized by a high, broad forehead; broad eyebrows with medial prominence and central sparseness; hypertelorism; large, deep-set, widely spaced eyes; and large, uplifted earlobes with a central depression, often described as “orecchiette” or “red blood cell–like.” Additional features include a saddle nose with a prominent, rounded tip and prominent columella; an open mouth with an M-shaped upper lip; and a pointed, triangular, narrow, and prominent chin (11, 18). In addition to the characteristic facial gestalt, individuals with MWS frequently present with craniofacial and oral abnormalities that may directly affect oral function and dental management. Patients with MWS often exhibit a highly arched palate, possibly secondary to hypotonia. Additional, less frequent craniofacial findings include bifid uvula, submucous cleft palate, cleft of the hard palate, and right unicoronal craniosynostosis (19). The facial phenotype of MWS is highly distinctive and tends to become more pronounced with age, thereby facilitating clinical recognition over time. Although individuals with MWS are frequently described as sociable, affectionate, and cheerful, behavioral and emotional dysregulation is also common (20). Frequently reported manifestations include anxiety, disruptive or antisocial behaviors, repetitive behaviors, oral self-stimulatory behaviors such as chewing objects or body parts, and reduced pain sensitivity (>50%) (21). Similarly to what has been reported in other populations with intellectual disabilities, individuals with MWS may experience substantial barriers in accessing and utilizing dental care services (22). Effective dentist–patient interaction relies heavily on the patient's ability to understand instructions, communicate discomfort, and cooperate during clinical procedures. However, expressive and receptive communication impairments may lead to increased anxiety, behavioral difficulties, and reduced compliance during dental visits (23, 24). Consequently, dental professionals may encounter difficulties in delivering appropriate care, resulting in unmet oral health needs and a higher prevalence of untreated oral conditions in this population (22). Furthermore, caregivers and healthcare providers may struggle to accurately recognize or interpret oral symptoms, thereby delaying diagnosis and treatment (25).

Autism spectrum disorder (ASD) is a neurodevelopmental disorder (26) characterized by persistent impairments in social communication and interaction, together with restricted and repetitive patterns of behavior, interests, or activities. ASD generally manifests before the age of three years and affects multiple developmental domains throughout life (27). Individuals with ASD share several clinically relevant features with patients affected by MWS, including intellectual disability, communication impairments, behavioral difficulties, and limited cooperation during dental care procedures. These factors are known to negatively influence access to oral healthcare and oral health outcomes. However, unlike MWS, ASD is not typically associated with syndrome-specific craniofacial dysmorphisms or distinctive oral manifestations (28). Furthermore, although ASD is considerably more prevalent and more widely recognized among healthcare professionals than MWS, substantial barriers to oral healthcare access persist in this population (29).

Despite growing interest in OHRQoL among pediatric populations with special healthcare needs, little is known about the oral health experience and its psychosocial impact in individuals with MWS. To the best of our knowledge, no published studies have specifically evaluated OHRQoL in individuals with Mowat-Wilson syndrome or compared it with that of other neurodevelopmental conditions. Assessing OHRQoL in children and adolescents with MWS may provide clinically relevant information that cannot be captured through conventional oral examinations alone. Given the frequent presence of severe intellectual disability, communication impairments, and complex medical needs, caregiver-reported outcomes may help identify difficulties affecting daily functioning, wellbeing, and family life that would otherwise remain unrecognized. Understanding these perceived impacts may contribute to a more comprehensive assessment of oral health needs and support the development of patient-centred dental care strategies for this vulnerable population. Therefore, the primary objective of this study was to evaluate caregiver-reported oral health-related quality of life (OHRQoL) in children and adolescents with Mowat-Wilson syndrome and compare it with peers with autism spectrum disorder (ASD) and neurotypical controls. Secondary exploratory objectives were to describe caregiver-reported functional limitations and patterns of dental care utilization across groups.

## 2 Material and methods

To address the research question, a cross-sectional questionnaire-based study was conducted. The research team was affiliated with the Department of Biomedical, Surgical and Dental Sciences, University of Milan, and the protocol was approved by the Ethics Committee Board of the University of Milan (approval date: 08 July 2025; approval number: 80/25) and conducted following the Declaration of Helsinki. The study protocol was prospectively registered at ClinicalTrials.gov (Identifier: NCT07476417). This study was reported in accordance with the STROBE (Strengthening the Reporting of Observational Studies in Epidemiology) Statement, and the STROBE checklist for cross-sectional studies was followed throughout the preparation of the manuscript.2.1 Inclusion and exclusion criteria

The inclusion criteria comprised parents/caregivers of children and adolescents aged between 6 and 18 years diagnosed with MWS, ASD, or without any known medical condition or neurodevelopmental disorder. In all groups, parents/caregivers were required to provide informed consent prior to accessing and completing the questionnaire. The exclusion criteria included children who were already participating in other studies or in oral health prevention programs.

### 2.1 Sample size

Recruitment and data collection for parents of MWS subjects were conducted in collaboration with the Italian Association Mowat–Wilson Onlus, a national organization supporting families of individuals diagnosed with MWS. An invitation to fill in an online questionnaire was distributed via email by the Association to all registered members (approximately 45 families with children of all ages), including detailed information about the study objectives. To reach caregivers not affiliated with the Association, recruitment was also promoted through online support groups, social media platforms, and informal caregiver networks. Given the rarity of MWS, no formal sample size calculation was performed; instead, all eligible subjects meeting the inclusion criteria during the study period were consecutively recruited. For each parent/guardian of a child with MWS enrolled as a case (*n* = 19), one participant with ASD and two neurotypical participants were selected as matched controls (ratio 1:1:2) from a larger database previously collected in a study by Salerno et al. (9). For each case, the pool of eligible controls was defined as all questionnaires in the source database matching the case for child's age. Controls already assigned to a previously processed case were excluded from subsequent eligible pools to prevent duplicate assignment across cases. Cases were processed in a randomized order to avoid systematic bias arising from progressive depletion of the eligible control pool. For each case, the required number of controls (one ASD and two neurotypical) was randomly selected without replacement from the corresponding eligible pool using the RAND() function in Microsoft Excel 2019 for Mac (Microsoft Corp., Redmond, WA, USA); random values were converted to static values before sorting, to ensure reproducibility of the selection. Matching between MWS and ASD participants was performed according to the child's age. Information on ASD severity level, intellectual and adaptive functioning, level of support needs, and specific sensory or motor profiles was not systematically collected in the source database and therefore could not be considered in the matching procedure. Data collection was conducted between November 2024 and September 2025.

### 2.2 Questionnaires

The short-form version of the Parental/Caregiver Perception Questionnaire (P-CPQ) and the Family Impact Scale Questionnaire (FIS) (30) were used. The P-CPQ consists of 16 items designed to assess children's oral health and its impact on overall well-being. The questionnaire is divided into four domains: Oral Symptoms, Functional Limitations, Emotional Well-being, and Social Well-being. Each item is scored using a five-point Likert scale, with responses coded as follows: “don't know” = 0, “never” = 1, “once or twice” = 2, “sometimes” = 3, “often” = 4, and “every day or almost every day” = 5. The total P-CPQ score is calculated by summing the scores of all items, yielding a possible range from 0 to 80. Higher scores indicate poorer oral health–related quality of life. The FIS questionnaire consists of 8 items assessing the impact of the child's oral health on the family's quality of life. It is divided into three domains: Family Activities, Parental Emotions, and Family Conflict. Responses are scored using the same five-point Likert scale adopted for the P-CPQ. The total FIS score is obtained by summing all item scores, resulting in a possible range from 0 to 40, with higher scores indicating poorer family oral health–related quality of life. Additional questions were included alongside the standardized questionnaires to collect personal and socioeconomic information regarding the respondent, as well as parental perceptions of the child's oral health and its impact on overall health. Parents' assessment of the child's oral health was rated using a five-point scale as follows: 1 = excellent, 2 = above sufficient, 3 = sufficient, 4 = insufficient, and 5 = poor. The perceived impact of oral health on the child's overall health was similarly evaluated using a five-point scale: 1 = not at all, 2 = a little, 3 = sufficiently, 4 = enough, and 5 = very much. The questionnaire had previously been validated in its Italian version (9). The P-CPQ and FIS are caregiver-reported instruments and were therefore considered suitable for use in populations with communication difficulties. However, although validated in pediatric populations, these instruments were not specifically developed for children with severe intellectual disability or profound communication impairment. Consequently, responses reflected caregiver perceptions and observations rather than direct patient reports.

### 2.3 Statistical analysis

All data obtained from the included questionnaires were entered into a spreadsheet (Excel™ 2024 for Windows; Microsoft Corp., Redmond, WA, USA) and subsequently processed for statistical analysis. Descriptive statistics were reported as mean ± standard deviation (SD) for continuous variables and as absolute and relative frequencies (n, %) for categorical variables. The normality of distribution of continuous variables was assessed using Shapiro–Wilk test, and homogeneity of variances among groups was evaluated using Levene's test. Based on these results, differences among the three groups (MWS, ASD, and neurotypical) were analyzed using one-way analysis of variance (ANOVA) for normally distributed variables with homogeneous variances, Welch's ANOVA for normally distributed variables with unequal variances, and the Kruskal–Wallis test for continuous variables not meeting the assumption of normality and for ordinal categorical variables. For nominal categorical variables with low expected cell counts, Fisher's exact test was used. When the Kruskal–Wallis test indicated a statistically significant overall difference among groups, pairwise *post-hoc* comparisons were performed using Dunn's test with Bonferroni correction to account for multiple comparisons. For variables analyzed using one-way ANOVA, significant differences were further explored using Tukey's honestly significant difference (HSD) *post-hoc* test. For variables analyzed using Welch's ANOVA, pairwise *post-hoc* comparisons were conducted using the Games–Howell test, which does not assume homogeneity of variances. Multiple linear regression analyses were performed to examine the association between study group and each outcome variable (P-CPQ, FIS, and TOT), while adjusting for participants' age, relationship to the child, and educational level. Three separate regression models were estimated, with P-CPQ, FIS, and TOT entered as the dependent variables. Robust standard errors (Huber–White estimator) were used to account for potential heteroscedasticity. Overall model significance was assessed using the *F*-test. Model fit was evaluated using the coefficient of determination (R^2^). Regression coefficients (*β*), together with their robust standard errors, t-values, 95% confidence intervals, and p-values, were reported to quantify the association between each predictor and the outcome variables. To assess multicollinearity among the independent variables, variance inflation factors (VIFs) were calculated. VIF values below commonly accepted thresholds (e.g., <5) were considered indicative of no relevant multicollinearity. A *p*-value <0.05 was considered statistically significant.

## 3 Results

Nineteen questionnaires completed by parents/caregivers of children with Mowat–Wilson syndrome were included in the present study and compared with 19 questionnaires completed by parents/caregivers of children with autism spectrum disorder and 38 questionnaires completed by parents/caregivers of neurotypical peers. The mean age of children was 10.86 ± 3.08 years. Demographic and socioeconomic characteristics of the children and their parents/caregivers are presented in Table 1. Regarding parental characteristics, the mean age of parents/caregivers who completed the questionnaire was 44.79 ± 5.96 years, with no statistically significant differences among the three groups (*p* = 0.621). Mothers were the most frequent respondents overall (85.53%), with a significantly higher proportion in the neurotypical control group compared with the MWS and ASD groups (*p* = 0.046). Most respondents (78.95%) had a high educational level (high school diploma or higher) and were regular workers (72.37%). Salary was the main source of family income in all groups (93.42%) and most families lived in owned houses (93.42%). Family income distribution was also similar across groups (*p* = 0.642), although the MWS group more frequently reported income between €25,001 and €40,000 (62.50%). No significant differences were observed in family income characteristics.

**Table 1:** Demographic, socioeconomic, and dental attendance characteristics of children with mowat-wilson syndrome, autism spectrum disorder, and neurotypical controls and their caregivers.

Variable	MWS	ASD	Neurotypical	*p*-value	Total
	*N* = 19	*N* = 19	*N* = 38		*N* = 76
Items related to family/parents					
Parent's age mean (SD)	44.21 (6.53)	45.95 (5.28)	44.50 (6.14)	0.621^a^	44.79 (5.96)
Parent/Guardian Status *n* (%)					
Father	5 (26.32)	4 (21.05)	2 (5.26)	**0**.**046**^b^	11 (14.47)
Mother	14 (73.68)	15 (78.95)	36 (94.74)		65 (85.53)
Educational level *n* (%)					
Middle school diploma	2 (10.53)	4 (21.05)	10 (26.32)	0.976^c^	16 (21.05)
High school diploma	9 (47.37)	4 (21.05)	7 (18.42)		20 (26.32)
University degree	6 (31.58)	10 (52.63)	18 (47.37)		34 (44.74)
Post-graduate qualifications	2 (10.53)	1 (5.26)	3 (7.89)		6 (7.89)
Type of house *n* (%)					
Property house	19 (100.00)	18 (94.74)	34 (89.47)	0.313^c^	71 (93.42)
Rented house	0 (0.00)	1 (5.26)	4 (10.53)		5 (6.58)
Employment status *n* (%)					
Regular worker	16 (84.21)	10 (52.63)	29 (76.32)	0.080^c^	55 (72.37)
Irregular worker	0 (0.00)	1 (5.26)	1 (2.63)		2 (2.63)
Unemployed	3 (15.79)	8 (42.11)	8 (21.05)		19 (25.00)
Source of family income *n* (%)					
Salary	18 (94.74)	16 (84.21)	37 (97.37)	0.168^c^	71 (93.42)
Accompanying allowance	0 (0.00)	2 (10.53)	1 (2.63)		3 (3.95)
Retiring allowance	1 (5.26)	1 (5.26)	0 (0.00)		2 (2.63)
Family income *n* (%)					
Less than € 15.000	2 (12.50)	3 (15.79)	2 (5.26)	0.642^c^	7 (9.59)
Between € 15.001 and € 25.000	1 (6.25)	5 (26.32)	10 (26.32)		16 (21.92)
Between € 25.001 and € 40.000	10 (62.50)	2 (10.53)	16 (42.11)		28 (38.36)
Between € 40.001 and € 60.000	0 (0.00)	6 (31.58)	5 (13.16)		11 (15.07)
Major than € 60.000	3 (18.75)	3 (15.79)	5 (13.16)		11 (15.07)
Item related to child					
Usual Dental setting *n* (%)					
Private dentist	4 (21.05)	6 (31.58)	34 (89.47)	**<0**.**001**^c^	16 (21.05)
Public dentist	10 (52.63)	9 (47.37)	0 (0.00)		47 (61.84)
None	5 (26.32)	4 (21.05)	4 (10.53)		13 (17.11)
Dental attendance *n* (%)					
Every 3 months	1 (5.26)	2 (10.53)	7 (18.42)		10 (13.16)
Every 6 months	7 (36.84)	8 (42.11)	12 (31.58)		27 (35.53)
Once a year	5 (26.32)	2 (10.53)	13 (34.21)		20 (26.32)
Less than once a year	1 (5.26)	1 (5.26)	1 (2.63)	0.398^c^	3 (3.95)
Only when necessary (e.g., pain)	1 (5.26)	2 (10.53)	2 (5.26)		5 (6.58)
He/she doesn't go to the dentist	4 (21.05)	4 (21.05)	3 (7.89)		11 (14.47)

N, number; SD, standard deviation; MWS, Mowat-Wilson syndrome; ASD, autism spectrum disorders. Statistically significant *p*-values (*p* < 0.05) are shown in bold.

a One-way ANOVA.

b Fisher's exact test.

c Kruskal-Wallis rank-sum test.

A statistically significant difference among groups was observed in the usual dental care setting (*p* < 0.001). Children with MWS and ASD relied more frequently on public dental services than neurotypical peers. In the MWS group, 10 children (52.63%) usually attended a public dental service, 4 (21.05%) a private dentist, and 5 (26.32%) had no usual dental care setting. A similar pattern was observed in the ASD group, where 9 children (47.37%) attended public dental services, 6 (31.58%) a private dentist, and 4 (21.05%) had no usual dental care setting. By contrast, most neurotypical children attended private dental services (*n* = 34, 89.47%), while only 4 children (10.53%) had no usual dental care setting. Dental attendance frequency did not differ significantly among groups (*p* = 0.398). However, irregular or absent dental attendance was descriptively more frequent among children with MWS and ASD than among neurotypical peers. In both the MWS and ASD groups, 4 children (21.05%) did not attend dental visits at all, compared with 3 children (7.89%) in the neurotypical group. When children attending dental visits only when necessary were also considered, irregular or absent attendance involved 5 children with MWS (26.32%), 6 children with ASD (31.58%), and 5 neurotypical children (13.16%).

Parents' perception of their child's oral health status across the three groups is presented in Table 2. Regarding parental perception of the child's oral health status, the highest mean score was observed in the ASD group (3.16 ± 0.76), compared with both the MWS group (2.74 ± 0.73) and the neurotypical group (2.45 ± 0.80). A statistically significant difference was found between the ASD and neurotypical groups (*p* = 0.006). Concerning the perceived impact of oral conditions on general well-being, children with MWS showed the highest mean score (3.21 ± 1.08), with statistically significant differences compared with both the ASD group (*p* = 0.019) and the neurotypical group (*p* = 0.005). No statistically significant difference was observed between ASD and neurotypical children (*p* = 1.000). Overall, while ASD children were reported to have poorer oral health status, the perceived impact of oral conditions on general wellbeing was greater in children with MWS.

**Table 2:** Caregiver-reported perceptions of oral health status and oral health impact on general wellbeing.

Variable	MWS	ASD	Neurotypical	*P* value			
	*N* = 19	*N* = 19	*N* = 38	MWS vs. ASD^b^	MWS vs. Neurotypical^b^	ASD vs. Neurotypical^b^	MWS vs. ASD vs. Neurotypical^a^
How would you classify the health of your child's teeth, lips, jaw and mouth?							
Mean ± SD	2.74 (0.73)	3.16 (0.76)	2.45 (0.80)	0.201	0.364	**0**.**006**	**0**.**015**
(1 = excellent; 2 = above sufficient; 3 = sufficient; 4 = insufficient; 5 = poor)							
How much was your child's general wellbeing influenced by the state of his teeth, lips, jaw or mouth?							
Mean ± SD	3.21 (1.08)	2.26 (1.41)	2.21 (1.17)	**0.019**	**0**.**005**	1.000	**0**.**008**
(1 = not at all; 2 = a little; 3 = sufficiently; 4 = enough; 5 = very)							

N, number; SD, standard deviation; MWS, Mowat-Wilson syndrome; ASD, autism spectrum disorders. Statistically significant *p*-values (*p* < 0.05) are shown in bold.

a Kruskal-Wallis rank-sum test.

b Dunn–Bonferroni's adjusted pairwise comparisons.

The mean scores for each item, each domain, and the total scores of the P-CPQ and FIS are presented in Table 3; *post hoc* pairwise comparisons was reported in Table 4. The mean total P-CPQ score was 37.11 ± 8.65 in the MWS group, 32.84 ± 8.85 in the ASD group, and 26.23 ± 5.05 among neurotypical group. In the Oral symptoms' domain, total scores were significantly higher in the MWS group than in the neurotypical group (*p* = 0.008), while no significant difference was observed between MWS and ASD or between ASD and neurotypical participants. Among individual items, halitosis and food impaction on the palate were significantly more frequent in the MWS group compared with both ASD and neurotypical groups (*p* = 0.030 and 0.002, respectively), whereas no significant group differences were observed for tooth or jaw pain and food impaction among teeth. The functional limitation domain showed marked group differences, with participants with MWS reporting significantly higher scores than both the ASD (*p* = 0.003) and neurotypical groups (*p* < 0.001). Difficulties in chewing solid food, mouth breathing, slower eating, and sleep disturbances were all significantly more frequent in the MWS group compared with neurotypical participants (*p* < 0.001), whereas differences between the ASD and neurotypical groups were limited. Individuals with ASD reported significantly higher levels of irritability and anxiety-related symptoms than neurotypical controls (*p* = 0.022 and 0.016, respectively), whereas differences involving the MWS group were not statistically significant for most emotional items. Similarly, in the social wellbeing, overall differences were observed between ASD and neurotypical participants (*p* = 0.041). The overall P-CPQ score was highest in the MWS group and was significantly greater than that of neurotypical participants (*p* < 0.001). The ASD group also showed significantly higher total P-CPQ scores than neurotypical controls (*p* = 0.016), whereas the difference between the MWS and ASD groups was not statistically significant. Total FIS score was significantly higher in families of ASD participants (21.58 ± 4.85) compared with both MWS (16.42 ± 7.09) (*p* = 0.016) and neurotypical participants (15.71 ± 5.11) (*p* = 0.001). Finally, the overall total score (P-PCQ + FIS) showed mean values of 53.53 ± 13.62 in the MWS group, 54.42 ± 12.60 in the ASD group, and 41.95 ± 8.93 in the neurotypical group. Significant differences were observed among groups, with both the MWS and ASD participants showing higher scores compared with neurotypical controls (*p* = 0.006 and 0.002, respectively), whereas no significant difference was found between the MWS and ASD groups.

**Table 3:** Caregiver-reported P-CPQ and FIS item scores, domain scores, and total scores in children with Mowat-Wilson syndrome, autism spectrum disorder, and neurotypical controls.

Variable	MWS	ASD	Neurotypical	*p*-Value	Total
	*N* = 19	*N* = 19	*N* = 38		*N* = 76
Oral symptoms	8.95 (3.26)	7.21 (2.86)	6.32 (1.66)	**0**.**008**^b^	7.20 (2.64)
Halitosis	2.89 (1.20)	2.05 (1.22)	1.82 (0.93)	**0**.**004**^c^	2.14 (1.14)
Tooth, mouth, lip or jaw pain	1.79 (0.79)	2.16 (1.26)	1.68 (0.87)	0.391^c^	1.83 (0.97)
Food stuck to the palate	2.11 (1.37)	1.11 (0.74)	1.0 (0.00)	**<0**.**001**^c^	1.30 (0.89)
Food stuck among teeth	2.16 (1.12)	1.89 (1.29)	1.82 (0.90)	0.531^c^	1.92 (1.05)
Functional limitation	12.21 (3.34)	7.63 (3.00)	5.68 (1.86)	**<0**.**001**^c^	7.80 (3.69)
Difficulty chewing solid food	3.37 (1.61)	1.47 (0.96)	1.13 (0.41)	**<0**.**001**^c^	1.78 (1.33)
Mouth breathing	3.0 (1.53)	1.68 (1.34)	1.45 (0.89)	**<0**.**001**^c^	1.89 (1.33)
Eating slower/Taking longer than others	2.84 (1.30)	2.11 (1.33)	1.5 (0.98)	**<0**.**001**^c^	1.99 (1.26)
Trouble sleeping	3.00 (1.45)	2.37 (1.16)	1.61 (0.92)	**<0**.**001**^c^	2.14 (1.25)
Emotional wellbeing	9.00 (2.96)	10.79 (2.85)	9.00 (2.49)	0.051^a^	9.45 (2.78)
Irritable or frustrated	2.74 (1.45)	3.00 (0.88)	2.29 (0.98)	**0**.**045**^c^	2.58 (1.12)
Sad	1.74 (0.81)	2.37 (1.07)	2.05 (0.87)	0.127^c^	2.05 (0.92)
Nervous, fearful or anxious	2.84 (1.01)	3.21 (0.86)	2.47 (0.89)	**0**.**035**^c^	2.75 (0.95)
Timid/embarrassed	1.68 (0.82)	2.21 (1.13)	2.18 (0.98)	0.159^c^	2.07 (0.99)
Social wellbeing	6.95 (3.24)	7.21 (3.31)	5.24 (1.58)	**0**.**048**^c^	6.16 (2.68)
He/She skipped school	1.79 (0.98)	1.84 (0.96)	1.37 (0.68)	0.079^c^	1.59 (0.85)
He/She avoided smiling in front of other children	1.37 (0.68)	1.16 (0.69)	1.11 (0.45)	0.061^c^	1.18 (0.58)
He/She had difficulty concentrating in class	2.63 (1.67)	2.26 (1.82)	1.61 (0.97)	0.076^c^	2.03 (1.45)
He/She didn't want to talk to other children	1.16 (1.34)	1.95 (1.39)	1.16 (0.59)	**0**.**036**^c^	1.36 (1.08)
P-CPQ	37.11 (8.65)	32.84 (8.85)	26.23 (5.05)	**<0**.**001**^b^	30.61 (8.43)
95% CI	32.94;41.27	28.58;37.11	24.58;27.90		28.68;32.53
Activity	4.11 (1.94)	5.58 (1.39)	3.16 (1.55)	**<0**.**001**^c^	4.00 (1.87)
Time off work	1.89 (1.05)	2.74 (1.05)	1.66 (0.91)	**0**.**002**^c^	1.99 (1.06)
Request of more attention	2.21 (1.03)	2.84 (1.46)	1.50 (0.86)	**<0**.**001**^c^	2.01 (1.20)
Emotion	9.89 (4.62)	12.32 (4.07)	8.61 (3.20)	**0**.**004**^a^	9.86 (4.06)
Less time for yourselves or for others	2.68 (1.45)	3.74 (1.15)	2.18 (1.11)	**<0**.**001**^c^	2.70 (1.35)
Disturbed sleep	2.84 (1.68)	2.89 (1.33)	2.13 (1.02)	0.057^c^	2.50 (1.31)
Feeling sad	2.16 (1.12)	3.05 (1.03)	2.24 (0.97)	**0**.**012**^c^	2.42 (1.07)
Feeling guilty	2.21 (1.27)	2.63 (1.42)	2.05 (0.96)	0.337^c^	2.24 (1.18)
Conflict	2.42 (1.71)	3.68 (0.95)	3.95 (1.63)	**0**.**002**^c^	3.5 (1.61)
Arguing with the child	1.37 (1.12)	2.58 (0.96)	2.37 (1.05)	**0**.**002**^c^	2.17 (1.13)
Blamed by your child	1.05 (0.78)	1.11 (0.57)	1.58 (0.82)	**0**.**009**^c^	1.33 (0.79)
FIS	16.42 (7.09)	21.58 (4.85)	15.71 (5.11)	**0**.**001**^a^	17.36 (6.06)
95% CI	13.00;19.84	19.24;23.91	14.03;17.39		15.97;18.74
P-CPQ + FIS	53.53 (13.62)	54.42 (12.60)	41.95 (8.93)	**<0**.**001**^b^	47.96 (12.60)
95% CI	46.96;60.09	48.35;60.50	39.01;44.88		45.08;50.84

N, number; SD, standard deviation; MWS, Mowat-Wilson syndrome; ASD, autism spectrum disorders; P-CPQ, Parental-Caregiver Perception Questionnaire; FIS, Family Impact Scale; CI: Confidence Interval. Statistically significant *p*-values (*p* < 0.05) are shown in bold.

a One-way ANOVA.

b Welch's one-way ANOVA.

c Kruskal-Wallis rank-sum test.

**Table 4:** *Post hoc* pairwise comparisons of caregiver-reported OHRQoL outcomes among study groups.

Variable	MWS vs. ASD	MWS vs. Neurotypical	ASD vs. Neurotypical
Oral symptoms^c^	0.202	**0**.**008**	0.429
Halitosis^a^	**0**.**030**	**0**.**002**	0.830
Tooth, mouth, lip or jaw pain	NS	NS	NS
Food stuck to the palate^a^	**<0**.**001**	**<0**.**001**	1.000
Food stuck among teeth	NS	NS	NS
Functional limitation^a^	**0**.**003**	**<0**.**001**	**0**.**046**
Difficulty chewing solid food^a^	**<0**.**001**	**<0**.**001**	0.568
Mouth breathing^a^	**0**.**005**	**<0**.**001**	0.904
Eating slower/Taking longer than others^a^	0.096	**<0**.**001**	0.138
Trouble sleeping^a^	0.332	**<0**.**001**	**0**.**031**
Emotional wellbeing^a^	0.111	1.000	0.055
Irritable or frustrated^a^	0.491	0.288	**0**.**022**
Sad	NS	NS	NS
Nervous, fearful or anxious^a^	0.411	0.299	**0**.**016**
Timid/embarrassed	NS	NS	NS
Social wellbeing^a^	1.000	0.113	**0**.**041**
He/She skipped school	NS	NS	NS
He/She avoided smiling in front of other children	NS	NS	NS
He/She had difficulty concentrating in class	NS	NS	NS
He/She didn't want to talk to other children^a^	**0**.**019**	0.550	0.072
P-CPQ^c^	0.302	**<0**.**001**	**0**.**016**
Activity^a^	**0**.**023**	0.099	**<0**.**001**
Time off work^a^	**0**.**033**	0.574	**0**.**001**
Request of more attention^a^	0.430	**0**.**024**	**<0**.**001**
Emotion^b^	0.130	0.455	**0**.**003**
Less time for yourselves or for others^a^	**0**.**025**	0.321	**<0**.**001**
Disturbed sleep	NS	NS	NS
Feeling sad^a^	**0**.**015**	1.000	**0**.**011**
Feeling guilty	NS	NS	NS
Conflict^a^	**0**.**007**	**<0**.**001**	1.000
Arguing with the child^a^	**0**.**002**	**0**.**003**	0.764
Blamed by your child^a^	0.978	**0**.**009**	**0**.**040**
FIS^b^	**0**.**016**	0.894	**0**.**001**
P-CPQ + FIS^c^	0.976	**0**.**006**	**0**.**002**

N, number; SD, standard deviation; MWS, Mowat-Wilson syndrome; ASD, autism spectrum disorders; P-CPQ, parental-caregiver perception questionnaire; FIS, family impact scale; NS, not significant. Statistically significant *p*-values (*p* < 0.05) are shown in bold.

a Dunn–Bonferroni's adjusted pairwise comparisons.

b Tukey's honestly significant difference test.

c Games–Howell test.

Multivariable linear regression analyses were reported in Table 5. For the P-CPQ model, the explained variance was moderate (R^2^ = 0.43). After adjustment, neurotypical participants showed significantly lower P-CPQ scores than children with MWS (*β* = −11.64, *p* < 0.001), whereas no significant difference was observed between the ASD and MWS groups. Child age and caregiver relationship were not independently associated with P-CPQ scores, while higher caregiver educational level was associated with lower P-CPQ scores. Similar findings were observed for the combined P-CPQ + FIS score (R^2^ = 0.36). In contrast, for the FIS model (R^2^ = 0.25), families of children with ASD reported significantly higher FIS scores than families of children with MWS (*β* = 5.75, *p* = 0.008), whereas no significant differences were observed between the MWS and neurotypical groups. Child age and caregiver relationship were not significantly associated with FIS scores, while higher caregiver educational level remained associated with lower family impact scores.

**Table 5:** Multivariable linear regression models assessing associations between study group and OHRQoL outcomes (P-CPQ, FIS, and combined P-CPQ + FIS scores).

	P-CPQ			
P-CPQ model: *n* = 76; F(30,37) = 9.94; *p* < 0.001; R-squared=0.43				
Reference categories: MWS; male; middle school diploma				

	Coeff	Robust SE	95% CI	*P*-value
ASD	−4.09	2.84	−9.76;1.58	0.155
Neurotypical	−11.64	2.21	−16.06;-7.23	**<0**.**001**
Parent's age	0.03	0.14	−0.24;0.31	0.814
Female	4.47	2.99	−1.49;10.43	0.139
High school diploma	−2.68	2.47	−7.62;2.25	0.282
University degree	−6.92	1.86	−10.63;−3.21	**<0**.**001**
Post-graduate qualifications	−5.39	1.98	−9.35;−1.43	**0**.**008**
FIS model: *n* = 76; F(30,37) = 4.06; *p* < 0.001; R-squared=0.25				
Reference categories: MWS; male; middle school diploma				
ASD	5.75	2.11	1.54;9.96	**0**.**008**
Neurotypical	−0.37	2.04	−4.44;3.69	0.855
Parent's age	0.01	0.11	−0.21;0.23	0.915
Female	0.96	2.17	−3.38;5.29	0.661
High school diploma	0.13	1.67	−3.20;3.46	0.938
University degree	−3.55	1.51	−6.56;−0.54	**0**.**021**
Post-graduate qualifications	−2.22	3.04	−8.29;3.85	0.468
P-CPQ + FIS model: *n* = 76; F(30,37) = 7.52; *p* < 0.001; R-squared=0.36				
Reference categories: MWS; male; middle school diploma				
ASD	1.66	4.35	−7.01;10.34	0.703
Neurotypical	−12.02	3.59	−19.18;−4.86	**0**.**001**
Parent's age	0.05	0.21	−0.38;0.47	0.833
Female	5.42	4.55	−3.66;14.51	0.238
High school diploma	−2.55	3.69	−9.91;4.81	0.491
University degree	−10.47	2.97	−16.41;−4.54	**0**.**001**
Post-graduate qualifications	−7.61	4.27	−16.14;0.92	0.079

N, number; CI, confidence interval; SE, standard error; ASD, autism spectrum disorders; P-CPQ, parental-caregiver perception questionnaire; FIS, family impact scale. Statistically significant *p*-values (*p* < 0.05) are shown in bold.

## 4 Discussion

This study evaluated caregiver-reported OHRQoL in children and adolescents with Mowat-Wilson syndrome and compared it with that of peers with ASD and neurotypical controls. The primary finding was that children with MWS showed significantly poorer OHRQoL than neurotypical peers, whereas overall OHRQoL did not significantly differ from that observed in children with ASD. These findings suggest that oral health-related impacts are more pronounced in MWS than in the general pediatric population, while presenting a burden comparable to that reported in another neurodevelopmental condition characterized by communication and behavioral challenges. ASD was therefore chosen as a clinically relevant neurodevelopmental comparison condition rather than as a disorder expected to be phenotypically equivalent to MWS. Children with ASD and MWS may share substantial support needs, communication and behavioral difficulties, and barriers to dental care, while differing in the presence of syndrome-specific craniofacial and oral features. This comparison may help distinguish oral health-related impacts associated with complex neurodevelopmental needs in general from functional difficulties that may be more specifically related to the MWS phenotype. Moreover, unlike MWS, ASD is relatively common, widely recognized by healthcare professionals, and supported by more established clinical and care pathways, providing a useful reference condition against which the unmet needs associated with a rare and less recognized syndrome can be contextualized.

Importantly, the pairwise comparisons indicated that the differences observed were not uniform across domains, supporting a more nuanced interpretation of the oral health experiences of children with MWS and ASD. Secondary exploratory analyses suggested distinct patterns of functional limitations and dental care experiences across groups. Although the overall burden of impaired OHRQoL was comparable between the MWS and ASD groups, the domains involved differed substantially, supporting the need to distinguish between different neurodevelopmental conditions when planning dental prevention and care.

The most prominent differences were observed within the functional limitation domain, suggesting that oral-health-related impacts in MWS may extend beyond conventional dental symptoms and involve broader oral functional challenges. The pairwise comparisons provide additional insight into these findings. In particular, children with MWS showed significantly greater difficulties in chewing solid food and mouth breathing than children with ASD, suggesting that these features may be more closely related to syndrome-specific characteristics of MWS than to neurodevelopmental impairment alone. By contrast, some differences, such as trouble sleeping, were primarily observed between MWS and neurotypical participants, indicating that not all functional limitations clearly distinguished MWS from ASD. In addition, halitosis and food impaction on the palate were more frequently reported in children with MWS. These findings suggest that the oral health burden in MWS is not limited to conventional dental problems, but includes a broader pattern of oral functional impairment. This is consistent with the known phenotype of MWS, which may include hypotonia, craniofacial dysmorphisms, highly arched palate, abnormal orofacial muscle function, and upper airway involvement, all of which can interfere with mastication, swallowing, breathing, and oral clearance (11, 18). Therefore, oral dysfunction may represent an under-recognized component of the MWS phenotype and an important source of unmet oral health need.

A further relevant finding was the discrepancy between parental perception of oral health status and the perceived impact of oral conditions on general wellbeing. Children with MWS did not show significantly poorer perceived oral health status than neurotypical peers, yet their caregivers reported the greatest impact of oral conditions on overall wellbeing. This suggests that even apparently moderate oral problems may have disproportionate consequences in children with MWS, possibly because of limited communication abilities, difficulties in expressing pain or discomfort, reduced adaptive coping skills, and the presence of complex medical comorbidities. Similar discrepancies between perceived oral health status and quality-of-life impact have previously been described in individuals with intellectual disabilities, highlighting how vulnerability and functional dependence may strongly influence OHRQoL outcomes (22, 23). In this context, caregiver observations become particularly important, as oral discomfort may be expressed through changes in eating, sleeping, behavior, or daily functioning rather than through verbal complaints.

A further finding emerged from the Family Impact Scale results. Although overall OHRQoL did not differ significantly between the MWS and ASD groups, families of children with ASD reported a significantly greater family burden. Children with ASD showed a different OHRQoL profile, characterized more by emotional, social, and family-related impacts than by oral functional impairment. The *post-hoc* analyses further refined this interpretation. While overall OHRQoL scores did not differ significantly between MWS and ASD, families of children with ASD reported significantly higher FIS scores. Similarly, ASD participants showed greater emotional and social impacts than neurotypical controls, whereas these differences were less evident for MWS. However, these findings should be interpreted carefully, as some emotional and social qu estionnaire items may be influenced by baseline characteristics of ASD itself, independently of oral health status. Higher scores for irritability, anxiety-related symptoms, and reluctance to interact with peers are consistent with the role of sensory sensitivity, behavioral dysregulation, and dental anxiety in ASD (24, 28). Families of children with ASD also reported the highest Family Impact Scale scores, reflecting greater caregiver burden. This finding is consistent with previous evidence showing that children with ASD and their families may experience substantial difficulties related to oral health, dental anxiety, communication barriers, and behavioral management during dental care (28). By contrast, families of children with MWS reported lower but still clinically relevant family impact, despite the greater functional impairment observed in the children. This may reflect differences in caregiver adaptation, expectations, coping strategies, and support networks, including patient associations. Overall, these findings suggest that children with MWS and ASD may share some access and communication barriers, but their unmet oral health needs are not identical.

Beyond OHRQoL outcomes, exploratory analyses of dental care utilization also revealed relevant differences among groups. Access to dental care also emerged as an important issue. Children with MWS and ASD more frequently relied on public dental services and more often lacked a usual dental care setting or regular dental attendance compared with neurotypical peers. Although the cross-sectional design does not allow causal interpretation, these findings point to possible access-related unmet dental care needs. This is consistent with the literature describing persistent structural, communicative, behavioral, and organizational barriers to dental care among individuals with intellectual disabilities and special healthcare needs (25). Similar barriers have been reported in a variety of populations with intellectual and developmental disabilities across different healthcare systems. Previous studies have described difficulties related to communication impairments, behavioural challenges, sensory sensitivities, limited availability of appropriately trained dental professionals, and reduced access to specialised dental services. These barriers have been reported not only in individuals with rare syndromes, but also among people with autism spectrum disorder and other developmental disabilities, suggesting that challenges in accessing dental care represent an internationally recognised issue rather than a phenomenon restricted to a specific healthcare system. This issue is particularly relevant in the Italian context, where most dental care is delivered privately and only part of dental assistance is covered by regional public health services. Unlike most medical specialties, dentistry is less consistently integrated into publicly funded care pathways, contributing to inequalities in access and to the under-prioritization of oral health at policy level (31). Strengthening public and hospital-based special care dentistry pathways may therefore be essential to reduce unmet needs in children with rare neurodevelopmental syndromes.

The multivariable regression analyses further supported these findings. After adjustment for caregiver and sociodemographic characteristics, neurotypical participants remained significantly associated with lower P-CPQ and combined P-CPQ + FIS scores compared with MWS participants, whereas ASD remained associated with a higher family impact burden. These results suggest that the observed differences were not fully explained by the demographic characteristics included in the models.

Overall, these findings suggest that oral health experiences in children with MWS involve multiple dimensions, including functional challenges, caregiver-perceived impacts, and potential barriers to dental care. These observations reinforce the importance of adopting a comprehensive and multidisciplinary approach when assessing and addressing oral health needs in this population. This last point deserves particular attention. Although the P-CPQ and FIS are validated and widely used instruments in OHRQoL research (6, 7), they were not specifically designed for children with intellectual disabilities or severe communication impairment. Some items, such as those addressing speaking, smiling, school concentration, social interaction, or emotional expression, may be difficult for caregivers to interpret in children whose baseline neurodevelopmental condition already affects these behaviors. Furthermore, the study population encompassed children and adolescents aged 6–18 years, representing different developmental stages. The functional, emotional, and social impacts captured by the questionnaires may vary according to age and developmental level, potentially influencing caregiver responses. Although the short-form P-CPQ and FIS have been used across this age range, age-related heterogeneity should be considered when interpreting the findings. Moreover, because MWS and ASD are associated with substantial differences in communication abilities, adaptive functioning, social interaction, and behaviour, some questionnaire responses may reflect characteristics of the underlying neurodevelopmental condition rather than oral-health-related impacts alone. In addition, ASD severity, intellectual and adaptive functioning, support needs, and specific sensory and motor profiles were not systematically assessed and were not used as matching criteria. Therefore, comparability between the MWS and ASD groups across these dimensions cannot be assumed. This is particularly relevant when interpreting individual P-CPQ domains, as differences in functional, emotional, or social items may partly reflect underlying neurodevelopmental, sensory, or motor characteristics rather than oral-health-related impacts alone. Consequently, between-group comparisons should be interpreted with caution, particularly for emotional and social domains. Similarly, caregivers may find it challenging to distinguish oral discomfort from behavioral manifestations related to the underlying syndrome. This limitation has been increasingly recognized in the field of oral health outcome measurement for persons with intellectual disabilities (32). Future research should therefore consider the development or adaptation of OHRQoL tools that include domains particularly relevant to children with complex neurodevelopmental conditions.

From a clinical perspective, these findings suggest that dental assessment in children with MWS should not be limited to caries detection and periodontal evaluation. A syndrome-informed approach should also include assessment of mastication, oral breathing, swallowing, oral motor function, palatal morphology, food retention, eating duration, caregiver-reported feeding difficulties, and barriers to daily oral hygiene. These aspects may require collaboration among paediatric dentists, orthodontists, speech and language therapists, nutrition specialists, paediatricians, geneticists, and rehabilitation professionals. Early and periodic dental referral within special care or hospital-based pathways may help identify functional problems before they lead to more severe oral or systemic consequences. Particular attention should be paid to mouth breathing, dysphagia-related symptoms, prolonged eating times, palatal food retention, and halitosis, as these problems were frequently reported by caregivers in the present study. Early recognition of these manifestations may reduce the risk of nutritional difficulties, choking episodes associated with retained food, sleep disturbances, and deterioration of oral hygiene. Practical management may include caregiver education regarding oral clearance after meals, monitoring of swallowing difficulties, and referral to speech and language therapists or multidisciplinary feeding teams when appropriate.

Several limitations should be acknowledged. First, the rarity of MWS resulted in a small sample size, limiting statistical power and generalizability. Nevertheless, given the low prevalence of the syndrome, the study sample represents an important and accessible group of participants with this rare condition, and the matched comparison with ASD and neurotypical controls strengthens the internal validity of the study.

The characteristics of the responding caregivers should also be considered when interpreting the generalizability of these findings. Mothers represented the majority of respondents across all study groups, a pattern that may partly reflect the persistent gender imbalance in family and caregiving responsibilities in Italy. Nevertheless, this predominance may introduce caregiver-related reporting bias, and the reported perceptions may not necessarily represent those of fathers or other caregivers. In addition, respondents generally had relatively high educational and socioeconomic characteristics. Although educational level and the main socioeconomic indicators did not differ significantly among the three study groups, the composition of the sample may limit the external generalizability of the absolute OHRQoL estimates to families with lower educational attainment or greater socioeconomic disadvantage. This is particularly relevant because caregiver educational level was independently associated with several OHRQoL outcomes in the multivariable analyses. However, the absence of significant socioeconomic differences among groups and adjustment for caregiver educational level in the multivariable models reduce the likelihood that these characteristics alone explain the observed between-group differences. Finally, given the rarity of MWS, recruitment relied on a limited population of eligible and accessible families, including participants reached through a patient association and caregiver networks. This recruitment strategy may have preferentially reached families who were more engaged with healthcare and support networks and should therefore be considered as an additional potential source of selection bias. Second, data were collected through parent-reported questionnaires and may therefore be influenced by subjective perception, recall bias, and caregiver stress. Third, the absence of standardized clinical oral examinations prevented the correlation of OHRQoL scores with objective oral findings, such as caries experience, periodontal status, occlusion, palatal morphology, xerostomia, or oral functional signs. Therefore, this study identifies caregiver-reported and perceived unmet needs but cannot quantify clinically diagnosed treatment needs. In addition, the broad age range included in the study and the use of caregiver-reported instruments not specifically designed for children with severe intellectual disabilities or communication impairments may have introduced additional heterogeneity in the assessment of OHRQoL outcomes. Finally, the cross-sectional design does not allow assessment of temporal changes or causal relationships between oral conditions, access to care, functional limitations, and quality of life.

In summary, children and adolescents with MWS showed a distinct profile of unmet oral health needs, mainly driven by oral functional limitations and by a high perceived impact of oral conditions on wellbeing. These needs differ from those observed in ASD and may not be fully captured by standard dental outcome measures. The findings support the development of syndrome-informed, multidisciplinary dental pathways that integrate oral function, caregiver-reported outcomes, and regular access to specialized dental care for children with rare neurodevelopmental disorders.

## 5 Conclusions

Children and adolescents with Mowat-Wilson syndrome showed poorer caregiver-reported OHRQoL than neurotypical peers. While overall OHRQoL was similar to that observed in ASD, differences emerged in caregiver-reported functional limitations. These findings describe caregiver-perceived oral health impacts and unmet needs rather than objectively assessed clinical oral conditions, highlighting the need for future studies combining OHRQoL measures with standardized clinical examinations.

## Data Availability

The raw data supporting the conclusions of this article will be made available by the authors, without undue reservation.
